# Nuclear and cytoplasmic p53 suppress cell invasion by inhibiting respiratory Complex-I activity via Bcl-2 family proteins

**DOI:** 10.18632/oncotarget.2320

**Published:** 2014-08-06

**Authors:** Eun Mi Kim, Jong Kuk Park, Sang-Gu Hwang, Wun-Jae Kim, Zheng-Gang Liu, Sang Won Kang, Hong-Duck Um

**Affiliations:** ^1^ Division of Radiation Cancer Biology, Korea Institute of Radiological & Medical Sciences, Seoul, Korea; ^2^ Division of Life and Pharmaceutical Science, Ewha Woman's University Seoul, Korea; ^3^ Department of Urology, College of Medicine, Chungbuk National University, Cheongju, Korea; ^4^ Center for Cancer Research, National Cancer Institute, National Institutes of Health, Bethesda, MD, USA

**Keywords:** p53, Bcl-2 family proteins, respiratory complex-I, cell invasion

## Abstract

Although the p53 tumor suppressor/transcription factor often accumulates in the cytoplasm of healthy cells, limited information is available on the cytoplasmic function of p53. Here, we show that cytoplasmic p53 suppresses cell invasion by reducing mitochondrial reactive oxygen species (ROS) levels. Analysis revealed that this function is mediated by Bcl-2 family proteins: Cytoplasmic p53 binds Bcl-w, liberating Bax, which then binds ND5, a subunit of respiratory complex-I, thereby suppressing complex-I activity and thus ROS production. The G13289A mutation of ND5, identified in cancer patients, prevents Bax/ND5 interactions and promotes ROS production and cell invasion. We also showed that Bcl-X_L_ and Bak can substitute for Bcl-w and Bax, respectively, regulating complex-I activity and supporting the cytoplasmic function of p53; nuclear p53 also suppresses complex-I activity by inducing Bax expression. Studies in animal models support the notion that p53 and Bcl-2 family proteins exhibit these functions *in vivo*. This study demonstrates a link between p53 and Bcl-2 proteins as regulators of ROS production and cellular invasiveness, and reveals complex-I, especially ND5, as their functional target.

## INTRODUCTION

The p53 tumor suppressor regulates numerous cellular functions, promoting cell death and suppressing cell migration and invasion [1]. Because p53 is a transcription factor, most studies of its mechanisms of action have focused on its transcriptional targets [2]. However, p53 wild-type and mutant derivatives frequently accumulate in the cytoplasm of normal and cancer cells [3-6], reflecting a non-transcriptional, cytoplasmic function in healthy cells. Nevertheless, information on this possibility is quite limited.

Bcl-2 family proteins are key regulators of cell death [7]. The pro-survival subfamily includes Bcl-2, Bcl-X_L_, and Bcl-w, whereas the pro-apoptotic subfamily comprises the multidomain (Bax and Bak) and BH3-only (Bid and others) groups. In healthy cells, pro-survival members bind to multidomain pro-apoptotic members and inhibit their apoptotic activities. However, these interactions are disrupted by BH3-only members, which are activated upon apoptotic stimulation. Multidomain pro-apoptotic members then attack the mitochondria, inducing apoptosis.

Recent evidence suggests that Bcl-2 proteins also regulate cellular invasiveness and thus cancer metastasis. For example, overexpression of Bcl-w, Bcl-X_L_, or Bcl-2 in various cancer cell types increased the migratory and invasive potential of these cells [8-14], and consistently facilitated the metastasis of cancer cells in animal models [15, 16]. Moreover, analyses of patient samples revealed the close association between up-regulation of pro-survival members and cancer metastasis [17, 18]. While the mechanisms underlying the new functions of Bcl-2 proteins are poorly understood, it has been shown that Bcl-w overexpression increases the levels of reactive oxygen species (ROS) in mitochondria, which, in turn, activates an invasion-promoting pathway that involves phosphoinositide 3-kinase (PI3K), Akt, and matrix metalloproteinase-2 (MMP-2) [8-10]. While the ability to increase ROS levels was also reported for Bcl-2 and Bcl-X_L_ [19, 20], Bax and Bak suppress cell invasion by reducing ROS levels [10]. Analysis of this functional antagonism has revealed that Bcl-w promotes ROS production by binding and neutralizing Bax suppression of ROS [10]. Therefore, to understand the mechanism by which Bcl-2 proteins regulate ROS production and cell invasion, we must discover how Bax suppresses ROS production.

In response to apoptotic stimulation, p53 translocates from the nucleus to the mitochondria, where it interacts with Bcl-2 proteins to promote apoptosis [21]. This supports the hypothesis that p53 constitutively accumulates in the cytoplasm of non-apoptotic, healthy cells, where it binds Bcl-2 proteins, and regulates ROS production and invasiveness. To investigate this possibility, we used cytoplasmic mutants of p53 and cells in which p53^wt^ accumulates in the cytoplasm. Bcl-w and Bax were used as prototypical pro-survival and multidomain pro-apoptotic members, respectively. Our data showed that nuclear and cytoplasmic p53 suppress ROS production and cell invasion. These effects were mediated by Bax, which was expressed through the transcriptional activity of nuclear p53, or released from Bcl-w upon binding of cytoplasmic p53 to Bcl-w. Interestingly, Bax suppressed ROS production by binding to ND5, a subunit of respiratory complex-I, and inhibiting complex-I activity. We also provide evidence supporting the hypothesis that p53 and Bcl-2 proteins perform these functions *in vivo*. Therefore, the p53/Bcl-2 proteins/complex-I pathway may be considered as a new therapeutic target for cancer metastasis.

## RESULTS

### Cytoplasmic p53 suppresses cell invasion

p53^K305N^ (a lysine-to-asparagine substitution at codon 305) has been reported to accumulate in the cytoplasm [22]. To confirm this, we expressed GFP-tagged p53^wt^ and p53^K305N^ in p53-null H1299 lung-cancer cells. DAPI (blue) and MitoTracker-Red were used to identify nuclei and mitochondria, respectively. Confocal microscopy revealed that the green signals in GFP-p53^wt^ transfectants became azure when merged with images of DAPI and MitoTracker-Red (Supplementary Figure S1). This was not observed in GFP-p53^K305N^-transfectants, where a significant portion of the green signal became yellow when the images were merged. These results support the nuclear and cytoplasmic (especially mitochondrial) localization of p53^wt^ and p53^K305N^, respectively, in this system.

**Figure 1:**
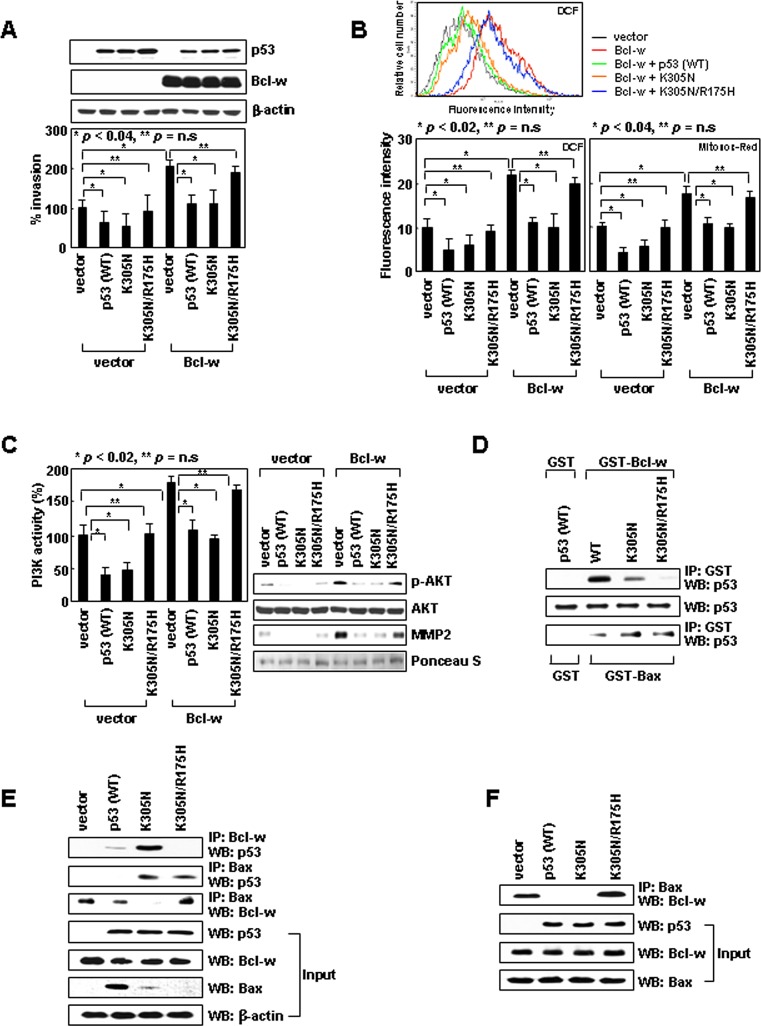
Cytoplasmic p53 suppresses cell invasion by binding to Bcl-w and liberating Bax from Bcl-w (A) Empty pcDNA3 or vectors encoding Bcl-w, p53, p53^K305N^, and p53^K305N/R175H^ were introduced into H1299 cells. Gene expression was confirmed by Western blotting with β-actin as a loading control. Invasiveness was assessed on Matrigel-coated filters. ns, not significant. (B) Transfectants were analyzed for cellular and mitochondrial ROS using DCF and MitoSOX Red probes, respectively. Flow cytometry profiles of key experiments are shown on top. (C) Lysates were prepared and immunoprecipitated with an anti-PI3K antibody. PI3K activity was analyzed by competitive ELISA. Lysates were also analyzed for Akt and phospho-Akt by Western blotting. Conditioned media were prepared and analyzed for MMP-2 levels by Western blotting. Protein loading for conditioned media was verified by Ponceau S staining. (D) Wild-type and mutant p53 proteins were prepared by *in vitro* translation and incubated with GST, GST-Bcl-w, or GST-Bax proteins. Precipitation was carried out with glutathione-coupled Sepharose beads. Precipitates and input controls were analyzed by Western blotting with anti-p53 antibody. (E) Lysates were immunoprecipitated with anti-Bcl-w or anti-Bax antibodies. Precipitates and input controls were analyzed by Western blotting. (F) Bax and Bcl-w, together with p53 or p53 mutants, were translated *in vitro*. The products were verified by Western blotting (input) and immunoprecipitated with anti-Bax antibody. Levels of Bcl-w in precipitates were compared.

Expression of p53^wt^ reduced cellular invasiveness (Figure 1A) in association with a decrease in ROS levels (Figure 1B), PI3K activity, Akt phosphorylation, and MMP-2 levels (Figure 1C). Similar results were obtained with p53^K305N^, suggesting nuclear and cytoplasmic p53 suppress cell invasion by blocking the ROS/PI3K/Akt/MMP-2 pathway. Since this pathway is stimulated by Bcl-w overexpression [10], we co-expressed Bcl-w with p53^wt^ or p53^K305N^, both of which abolished the ability of Bcl-w to activate the invasion pathway (Figure 1A-C). Similar results were obtained when the experiments were performed with H460 lung cancer cells (Supplementary Figure S2). Therefore, both nuclear and cytoplasmic p53 appear to antagonize the invasion-promoting action of Bcl-w in multiple cell types. The introduction of p53^wt^, p53^K305N^, and/or Bcl-w did not significantly influence cellular viability under these (Supplementary Figure S3) and other experimental conditions [23], suggesting their effects on cell invasion do not reflect alterations in cellular viability.

### p53^K305N^ binds Bcl-w and disrupts its interaction with Bax

To investigate the mechanisms underlying p53 activity, we first focused on cytoplasmic p53. The mitochondrial localization of p53^K305N^ suggests it may suppress Bcl-w-induced invasion by binding to Bcl-w. Co-immunoprecipitation assays using recombinant proteins (Figure 1D) or cell lysates (Figure 1E) indeed supported this interaction. The R175H mutation (an arginine-to-histidine substitution at codon 175) in p53, which abolishes Bcl-2 and Bcl-X_L_ binding [24], was introduced into p53^K305N^ (p53^K305N/R175H^). This double mutant was cytoplasmic, and a significant proportion was associated with mitochondria (Supplementary Figure S1). However, in contrast to p53^K305N^, p53^K305N/R175H^ neither interacted with Bcl-w (Figure 1D and E) nor reversed Bcl-w-induced signaling and cell invasion (Figure 1A-C). This suggests that Bcl-w binding is required for cytoplasmic p53 to antagonize the invasion-promoting action of Bcl-w. p53^K305N/R175H^ also failed to reduce the invasiveness of control cells, which do not overexpress Bcl-w (Figure 1A-C), supporting the involvement of endogenous Bcl-w in the cytoplasmic p53-mediated reduction in the basal invasiveness of H1299 cells.

Bcl-w promotes cell invasion by binding to Bax [10]; thus, binding of p53 to Bcl-w might cause dissociation of the pre-existing Bcl-w/Bax complex. This complex indeed disappeared in the presence of p53^K305N^, but not p53^K305N/R175H^ (Figure 1E and F). These findings suggest cytoplasmic p53 suppresses cell invasion by binding to Bcl-w and liberating Bax. p53^K305N^ also interacted with Bax (Figure 1D and E); however, in contrast to Bcl-w, Bax interacted with p53^K305N/R175H^ as well, suggesting that residue 175 of p53 is critical for binding to Bcl-w, but not Bax. Considering that only p53^K305N^, but not p53^K305N/R175H^, suppressed cell invasion despite the near-equal binding of these mutants to Bax, p53 binding to Bax does not appear to influence cellular invasiveness.

### Bax binds to complex-I, suppressing its activity and ROS production

**Figure 2:**
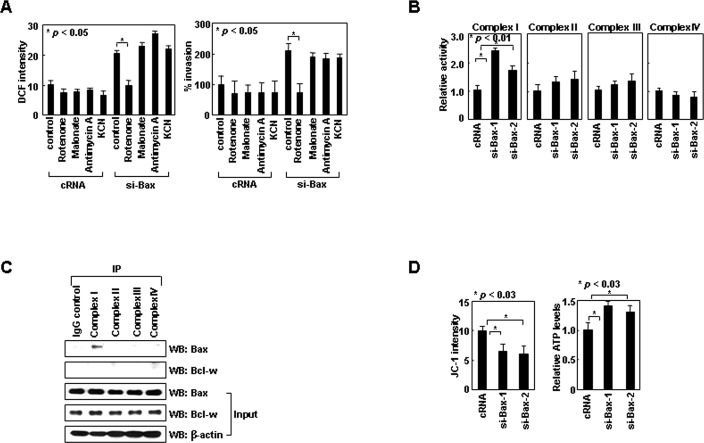
Bax inhibits ROS production by binding to complex-I (A) Control or Bax siRNA was introduced into H1299 cells. After 24 h, ROS levels and invasiveness were assessed in the presence or absence of rotenone (1 μM), malonate (5 mM), antimycin A (10 μM), or KCN (500 μM). (B) Cells treated with control or two sets of Bax siRNA were lysed and analyzed for complex I-IV activities. (C) H1299 cell lysates were immunoprecipitated using antibodies against each complex. Levels of Bax and Bcl-w in precipitates and input controls were compared. A control IgG, used as a negative control, is shown in the first lane. (D) Cells treated with siRNA were loaded with JC-1 and analyzed for ΔΨ_m_ by flow cytometry. Alternatively, cells were lysed for ATP assays.

Bax promotes ROS production in apoptotic cells by inducing mitochondrial permeability transition (MPT) [25]. However, the MPT inhibitor cyclosporine A at concentrations that inhibited the ROS production induced by lethal doses (10 Gy) of γ-rays (Supplementary Figure S4A), failed to reduce the ROS production induced by siRNA-mediated knockdown of Bax (Supplementary Figure S4B). Thus, Bax may regulate ROS production via different mechanisms in apoptotic and healthy cells. To determine how Bax suppresses ROS production in healthy cells, we focused on the mitochondrial respiratory chain, a major source of ROS in healthy cells [26]. The chain is composed of four multimeric protein complexes (complex I-IV). Rotenone, an inhibitor of complex-I, abolished the induction of ROS accumulation and cell invasion following Bax knockdown (Figure 2A). This effect was not observed with malonate, antimycin A, or KCN, which inhibit complexes II, III, and IV, respectively. These results suggest the specific role of complex-I in Bax knockdown-induced ROS accumulation. Bax knockdown consistently increased the activity of complex-I, but not that of other complexes (Figure 2B). Moreover, Bax was co-immunoprecipitated only with complex-I (Figure 2C). These data suggest Bax binds to complex-I and inhibits its activity, which results in a reduction in ROS production and cellular invasiveness. Bax knockdown also increased the ΔΨ_m_ and cellular ATP levels (Figure 2D), suggesting inhibition of complex-I activity by Bax results in a decrease in the ΔΨ_m_ and thus ATP production.

### The C-terminal tail of Bax binds to ND5

**Figure 3:**
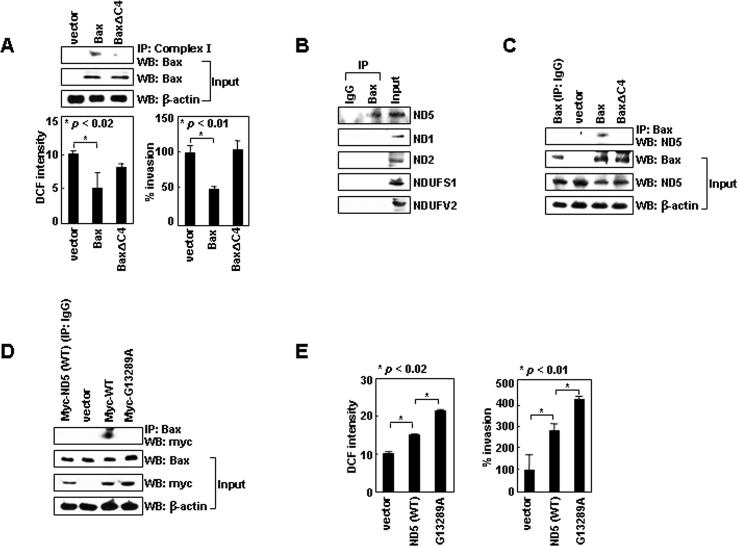
Bax binds to ND5 via its C-terminal tail (A) LoVo cells transfected with pTRE, pTRE-Bax, or pTRE-BaxΔC4 vectors were treated with tetracycline (1 μg/mL) for 16 h to induce gene expression. Lysates were prepared and immunoprecipitated with anti-complex-I antibody. Precipitates and inputs were analyzed by Western blotting with anti-Bax antibody. Transfectants were also analyzed for ROS levels and invasiveness. (B) H1299 lysates were immunoprecipitated with anti-Bax or control IgG. Levels of ND5, ND1, ND2, NDUFS1, and NDUFV2 in precipitates and inputs were assessed by Western blotting. (C) LoVo cell transfectants were lysed and immunoprecipitated with anti-Bax or control IgG. Levels of ND5 were compared. (D) H1299 cells transfected with empty pCMV/myc/mito vectors or vectors encoding ND5 and ND5^G13289A^ were lysed. Immunoprecipitation and Western blotting were performed. (E) The transfectants were assayed for ROS and invasiveness.

Bax can exist in an anchored form, wherein its C-terminal tail is inserted into the outer mitochondrial membrane in unstressed cells [27-30]. This topology allows the four C-terminal residues of Bax (KKMG) to protrude into the intermembrane space. Human complex-I consists of 45 subunits that form two arms; a membrane arm, which resides in the inner mitochondrial membrane, and a matrix arm, which protrudes into the mitochondrial matrix [31]. Some of the subunits in the membrane arm are exposed to the intermembrane space; thus, the C-terminal four residues of Bax might be involved in the interaction of Bax with complex-I. To investigate this possibility, we deleted these residues (BaxΔC4) and expressed Bax and BaxΔC4 using an inducible vector in LoVo colon-cancer cells, which do not express endogenous Bax [32]. This treatment did not significantly influence viability (Supplementary Figure S5). Whereas expression of Bax resulted in efficient formation of a complex between Bax and complex-I, complex formation was diminished in BaxC4-transfectants (Figure 3A). This suggests that the C-terminal four residues support binding of Bax to complex-I. Moreover, cellular ROS levels and invasiveness were reduced by the expression of Bax, but not BaxΔC4, supporting the requirement of Bax/complex-I interaction for the ROS-suppressing function of Bax.

To determine which part of complex-I interacts with Bax, we analyzed three subunits in the membrane arm (ND1, ND2, ND5) and two subunits in the matrix arm (NDUFS1, NDUFV2) and found that Bax co-immunoprecipitated only with ND5 (Figure 3B and C). However, ND5 did not co-immunoprecipitate with BaxΔC4 (Figure 3C), suggesting that Bax specifically binds to ND5 through its C-terminal tail.

### A natural mutation of ND5 prevents binding to Bax

Interestingly, complex-I, especially ND5, is a frequent target for natural mutation in lung cancer patients [33]. The G13289A mutation of ND5 is one such example. In contrast to ND5, ND5^G13289A^ failed to bind to Bax (Figure 3D). Moreover, while the overexpression of ND5 resulted in an increase in ROS production and cellular invasiveness, ND5^G13289A^ was superior to ND5 in inducing such effects (Figure 3E). These results support the notion that ND5 promotes ROS production and cell invasion, and this activity is suppressed by binding to Bax.

### Bcl-w activates complex-I by inhibiting Bax/complex-I interactions

**Figure 4:**
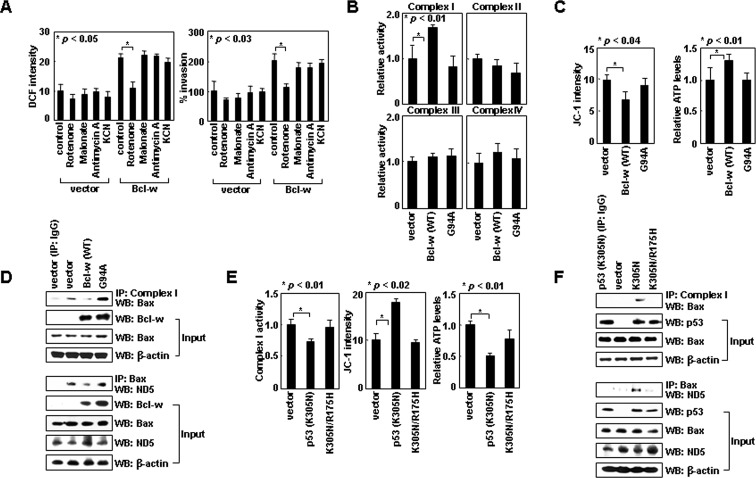
Bcl-w and cytoplasmic p53 regulate the interaction of Bax with complex-I (A) Control and Bcl-w transfectants of H1299 cells were analyzed for ROS levels and invasiveness in the presence or absence of rotenone (1 μM), malonate (5 mM), antimycin A (10 μM), or KCN (500 μM). (B) Control, Bcl-w, and Bcl-w^G94A^ transfectants were lysed and complex I-IV activities were compared. (C) Transfectants were analyzed for ΔΨ_m_ and ATP. (D) Cells were lysed, immunoprecipitated, and analyzed by Western blotting. (E) Control, p53^K305N^, and p53^K305N/R175H^ transfectants were analyzed for complex-I activity, ΔΨ_m_, and ATP. (F) The transfectants were lysed, immunoprecipitated, and analyzed by Western blotting.

Like Bax siRNA, Bcl-w-induced ROS accumulation and cell invasion were also prevented specifically by rotenone (Figure 4A). Bcl-w overexpression consistently elevated the activity of complex-I, but not that of complexes II-IV (Figure 4B), and increased ΔΨ_m_ and ATP levels (Figure 4C). Therefore, Bcl-w appears to activate complex-I, resulting in increases in ΔΨ_m_ and ATP levels as well as ROS production and cellular invasiveness. However, in contrast to Bax, Bcl-w did not efficiently bind to any respiratory complexes (Figure 2C), raising the possibility that Bcl-w activates complex-I by binding to Bax. Indeed, Bcl-w failed to increase complex-I activity, Δ_m_, and ATP levels when its interaction with Bax was prevented by introducing the G94A mutation into Bcl-w [10] (Figure 4B and C). Moreover, Bax/complex-I and Bax/ND5 interactions were dramatically reduced by overexpression of Bcl-w, but not Bcl-w^G94A^ (Figure 4D), suggesting that Bcl-w prevents Bax/complex-I (ND5) interactions by binding to Bax. Thus, it appears that Bcl-w activates complex-I by binding to Bax and promoting the release of Bax from complex-I.

### Cytoplasmic p53 suppresses complex-I activity by promoting Bax/complex-I interactions

These findings suggest cytoplasmic p53 also regulates complex-I activity. p53^K305N^ expression indeed reduced complex-I activity, and thus ΔΨ_m_ and ATP levels as well (Figure 4E). In addition, p53^K305N^ expression promoted Bax/complex-I and Bax/ND5 interactions (Figure 4F), suggesting cytoplasmic p53 suppresses complex-I activity by facilitating Bax/complex-I interactions. This activity may be mediated by binding to Bcl-w, which dissociates the Bcl-w/Bax complex. Indeed, p53^K305N/R175H^ failed to mimic the actions of p53^K305N^ (Figure 4E and F), supporting the requirement of p53/Bcl-w interaction for Bax/complex-I interaction and inhibition of complex-I activity.

### Cytoplasmic p53^wt^ mimics p53^K305N^

**Figure 5:**
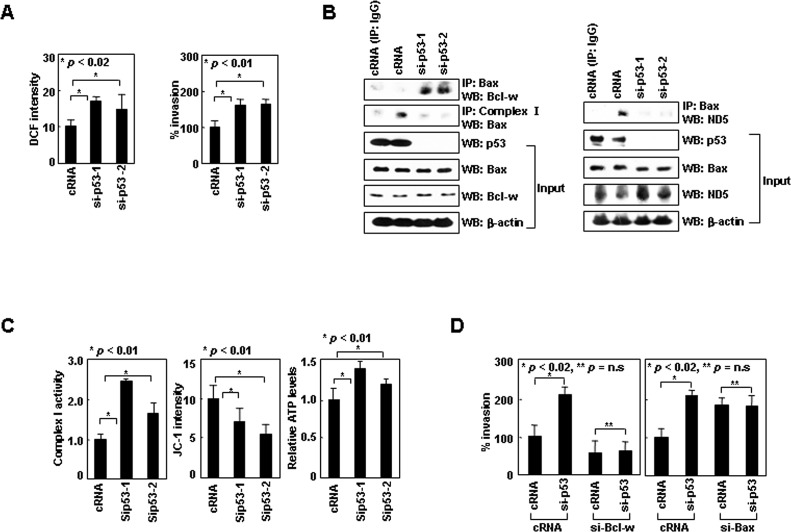
Cytoplasmic p53^wt^ mimics p53^K305N^ (A) IMR-32 cells treated with control or p53 siRNA were analyzed for ROS and invasiveness. (B) Lysates were immunoprecipitated with anti-Bax or anti-complex-I antibodies. Precipitates and input controls were analyzed by Western blotting. (C) Treated cells were analyzed for complex-I activity, ΔΨ_m_, and ATP. (D) IMR-32 cells were treated with control, p53, Bcl-w, and Bax siRNAs for 24 h and assayed for invasiveness.

To validate the functions of cytoplasmic p53, we used human IMR-32 neuroblastoma cells in which p53^wt^ accumulates in the cytoplasm for unknown reasons [34]. Knockdown of p53 in these cells by siRNA increased ROS levels and cellular invasiveness (Figure 5A), suggesting cytoplasmic p53^wt^ suppresses ROS-dependent invasion in these cells. p53 knockdown greatly increased Bcl-w/Bax complex levels without significant influence on Bcl-w or Bax levels (Figure 5B). This was accompanied by a decrease in Bax binding to complex-I and ND5 (Figure 5B), as well as increases in complex-I activity, ΔΨ_m_, and ATP levels (Figure 5C). These results suggest cytoplasmic p53^wt^ facilitates the dissociation of Bax from Bcl-w, resulting in the association of Bax with complex-I (ND5) and thus a reduction in complex-I activity, ΔΨ_m_, and ATP levels. Moreover, p53 knockdown with concurrent siRNA-mediated knockdown of Bcl-w or Bax failed to alter cellular invasiveness (Figure 5D), suggesting cytoplasmic p53^wt^ suppresses cell invasion in a manner dependent upon Bcl-w and Bax. Overall, the characteristics of cytoplasmic p53^wt^ are similar to those of p53^K305N^, suggesting both suppress cell invasion via the same mechanism.

### Bcl-X_L_ and Bak can substitute for Bcl-w and Bax, respectively, in support of the cytoplasmic functions of p53

**Figure 6:**
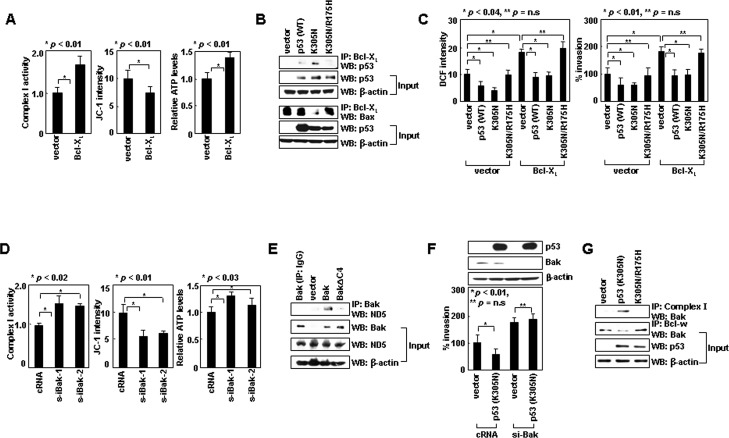
Bcl-X_L_ and Bak can substitute for Bcl-w and Bax, respectively, in regulating complex-I (A) Control and Bcl-X_L_ transfectants of H1299 were analyzed for complex I activity, ΔΨ_m_, and ATP. (B) Control, p53, p53^K305N^, and p53^K305N/R175H^ transfectants of H1299 cells were lysed and immunoprecipitated with anti-Bcl-X_L_ antibody. Precipitates were then analyzed by Western blotting with anti-p53 and anti-Bax antibodies. (C) Cells were analyzed for ROS and invasiveness. (D) LoVo cells were treated with control or Bak siRNA for 24 h and analyzed for complex I activity, ΔΨ_m_, and ATP. (E) LoVo cells were transfected with empty pTRE vector or pTRE expression vectors for Bak or BakΔC4. Transfectants were treated with tetracycline (1 μg/mL) for 24 h to induce gene expression. Lysates were prepared and immunoprecipitated with anti-Bak antibody. The levels of ND5 were analyzed by Western blotting. (F) Control or Bak siRNA as well as pcDNA3 or pcDNA3/p53^K305N^ were introduced into LoVo cells in the indicated combinations and invasiveness was compared. (G) Control, p53^K305N^, and p53^K305N/R175H^ transfectants of LoVo cells were lysed and immunoprecipitated with anti-complex-I or anti-Bcl-w antibodies. Bak levels were assessed by Western blotting.

The role of Bcl-w and Bax in the cytoplasmic function of p53 may extend to other pro-survival and multidomain pro-apoptotic members. Like Bcl-w, Bcl-X_L_ overexpression increased complex-I activity, ΔΨ_m_, and ATP levels (Figure 6A). Moreover, Bcl-X_L_ bound to p53^K305N^, but not p53^K305N/R175H^, and only p53^K305N^ antagonized the ability of Bcl-X_L_ to interact with Bax (Figure 6B) and increase ROS levels and cellular invasiveness (Figure 6C). These results suggest binding of cytoplasmic p53 to Bcl-X_L_ promotes the release of Bax from Bcl-X_L_, which then results in suppression of complex-I activity, ROS production, and cellular invasiveness. Therefore, Bcl-X_L_ and Bcl-w appear to play common roles in the invasion-suppressing action of cytoplasmic p53.

To determine whether Bak could mimic Bax in this system, we used LoVo cells, in which Bak-knockdown increased ROS levels and cellular invasiveness [10]. These effects were accompanied by increases in complex-I activity, ΔΨ_m_, and ATP (Figure 6D). Moreover, Bak interacted with ND5 in a manner that depended on its C-terminal 4 residues (FFKS) (Figure 6E). These results suggest that Bak, like Bax, binds via its C-terminal tail to ND5, and suppresses complex-I activity, ROS production, and cellular invasiveness. Moreover, whereas the introduction of p53^K305N^ reduced the invasiveness of control LoVo cells, this effect was not observed in Bak-knockdown LoVo cells (Figure 6F), suggesting that p53^K305N^ suppresses LoVo cell invasion in a Bak-dependent manner. The introduction of p53^K305N^ indeed facilitated dissociation of Bak from Bcl-w, and enhanced the binding of Bak to complex-I (Figure 6G). These effects were not observed with p53^K305N/R175H^, suggesting cytoplasmic p53 binds to Bcl-w to liberate Bak. The properties of Bak are similar to those of Bax, suggesting both suppress cell invasion via a common mechanism.

### Nuclear p53 suppresses invasion by inducing Bax expression

**Figure 7:**
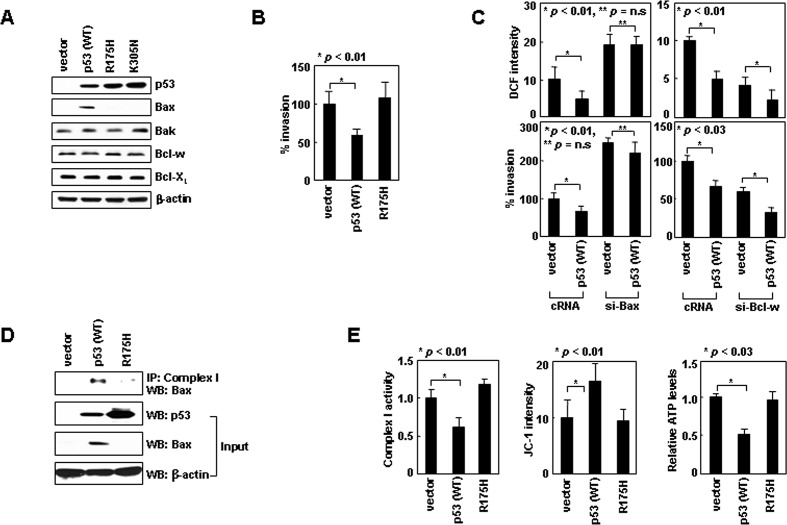
Nuclear p53 suppresses cell invasion by inducing Bax expression (A) p53 wild-type or mutant derivatives were expressed in H1299 cells. Protein expression was assessed by Western blotting. (B) Invasion assays. (C) Cells transfected with siRNAs and expression vectors were analyzed for ROS and invasiveness. (D) Transfectants were lysed, immunoprecipitated, and analyzed by Western blotting. (E) Transfectants were analyzed for complex-I activity, ΔΨ_m_, and ATP.

To investigate the mechanism whereby nuclear p53 suppresses invasion, we focused on Bax, a transcriptional target of p53. Expression of p53 in H1299 cells indeed elevated the levels of Bax, but not Bak, Bcl-w, or Bcl-X_L_ (Figure 7A). The Bax induction was not observed by expressing p53^R175H^, which localized to the nucleus (Supplementary Figure S1), but lacked DNA-binding ability [35]. p53^R175H^ also failed to suppress invasion (Figure 7B), suggesting that the transcriptional activity of nuclear p53 and subsequent Bax accumulation are required for this activity. The requirement of Bax was indeed supported by data showing that p53 failed to reduce ROS and invasiveness when Bax accumulation was prevented by siRNA (Figure 7C). In contrast, nuclear p53 efficiently reduced ROS levels and cellular invasiveness even when Bcl-w was knocked down. This suggests that nuclear p53 suppresses invasion in a manner independent of Bcl-w, which is consistent with the notion that Bax acts downstream of Bcl-w. p53 expression also facilitated Bax/complex-I interactions (Figure 7D), and thus reduced complex-I activity, ΔΨ_m_, and ATP levels (Figure 7E). These effects were not observed with p53^R175H^, indicating a requirement for p53 transcriptional activity. These data suggest nuclear p53 suppresses invasion by inducing Bax, which then binds to complex-I and inhibits ROS production.

### The p53/Bcl-w/Bax system regulates cancer cell intravasation

**Figure 8:**
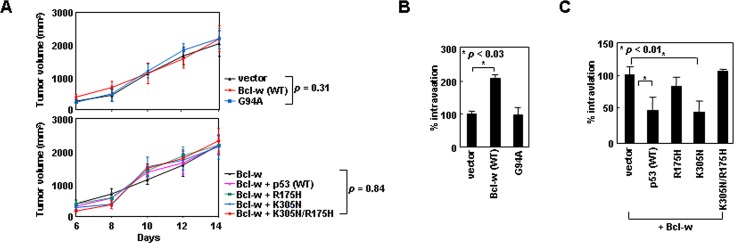
Cytoplasmic p53 and Bcl-w regulate cancer cell intravasation via mechanisms that depend on binding to Bcl-w and Bax, respectively (A) H460 cells transfected with empty pEGFP-C1 vectors or the vectors encoding the indicated constructs were implanted to form xenograft tumors in mice. Tumor volumes were measured on the indicated days after implantation. After 2 weeks, mice were photographed and blood was obtained. (B and C) Blood cells were stained with DAPI and analyzed by confocal microscopy. Circulating tumor cells were identified as GFP- and DAPI-positive cells. Photographs of experimental mice and confocal images of analyzed cells are presented in Supplementary Figure S6.

To investigate whether the p53/Bcl-w/Bax system modulates cellular behavior *in vivo*, we utilized a mouse model to compare the intravasation potential of cancer cells, a property that depends on their invasive activity [36]. We established xenograft tumors in mice by using H460 lung cancer cells transfected with GFP-expressing vectors encoding the wild-type or mutant derivatives of Bcl-w and p53. Expression of these exogenous genes did not significantly influence the size of xenograft tumors (Figure 8A). Tumor formation was followed by the appearance of the GFP-expressing tumor cells in mouse blood. The number of these circulating cells was increased by overexpression of Bcl-w, but not Bcl-w^G94A^ (Figure 8B), suggesting Bcl-w promotes the intravasation of tumor cells through binding to Bax. This intravasation-promoting ability of Bcl-w was antagonized by expression of exogenous p53 or p53^K305N^, but not by p53^R175H^ or p53^K305N/R175H^ (Figure 8C), suggesting that nuclear and cytoplasmic p53 antagonize Bcl-w-induced tumor cell intravasation through transcriptional activity and Bcl-w binding, respectively. The properties of p53 and Bcl-w are similar to those determined in cell culture. Therefore, regulation of tumor cell invasion by the p53/Bcl-w/Bax system appears to be applicable *in vivo*.

## DISCUSSION

**Figure 9:**
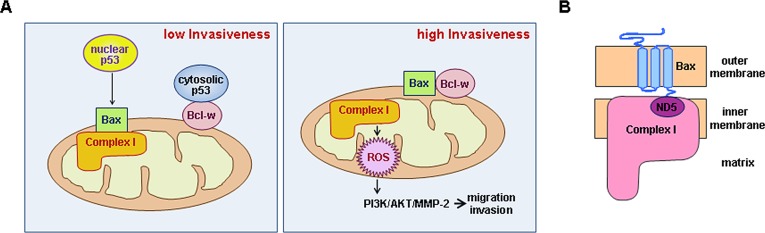
Schematic models (A) *Left:* Cytoplasmic p53 binds Bcl-w, promoting release of Bax and allowing its interaction with complex-I. This inhibits complex-I activity and decreases ROS production and cellular invasiveness. Nuclear p53 also facilitates Bax/complex-I interactions by promoting Bax expression. *Right:* Up-regulation of Bcl-w or down-regulation of p53 promotes Bcl-w/Bax interactions, releasing Bax from complex-I. This up-regulates complex-I activity and increases ROS production. ROS then stimulate the PI3K-dependent invasion pathway. (B) Interaction of Bax with complex-I. Bax resides in the outer mitochondrial membrane as a tail-anchored form, protruding its C-terminal tail into the inter-membrane space. This topology may allow interaction of the tail with ND5 in the membrane arm of complex-I.

We have shown that p53 suppresses invasion not only by acting in the nucleus but also through its actions in the cytoplasm. Our data suggest this novel function operates via the following mechanism: Cytoplasmic p53 binds to Bcl-w, which facilitates dissociation of Bax from Bcl-w. Bax then binds to complex-I and inhibits its activity. This results in a decrease in ROS production and invasiveness. However, when Bcl-w is up-regulated or the levels of cytoplasmic p53 are relatively low, Bcl-w/Bax interactions increase, promoting Bax release from complex-I. This leads to an increase in complex-I activity, and thus ROS production and invasiveness. Given that Bcl-X_L_ and Bak can substitute for Bcl-w and Bax, respectively, in this system, cytoplasmic p53 appears to suppress complex-I activity by inhibiting the interactions between pro-survival and multidomain pro-apoptotic Bcl-2 proteins. We have further verified that nuclear p53 can also suppress complex-I activity by inducing Bax expression. Collectively, our data suggest that p53 inhibits the mitochondrial pathway of cell invasion in a transcription-dependent and transcription-independent manner, summarized schematically in Figure 9A. Our findings are consistent with previous reports that although p53 promotes ROS production in apoptotic cells [37], it decreases ROS levels in healthy cells in association with decreased cellular energy metabolism and mTOR signaling or increased cellular antioxidant capacity [38-40]. Importantly, our model may operate *in vivo*, as we have shown that p53 and Bcl-w regulate the intravasation of cancer cells in an animal model. Given our findings, p53 appears to contribute to the suppression of tumor progression even through actions in the cytoplasm. Previous reports have also shown that cytoplasmic accumulation of p53 supports the survival of endometrial carcinoma patients as much as its nuclear localization [41].

Another important finding is that complex-I is a major target of Bcl-2 proteins in the regulation of mitochondrial metabolism and ROS production. Consistent with the pro-oxidant role of pro-survival Bcl-2 members in healthy cells, Bcl-2 and Bcl-X_L_ have been shown to increase mitochondrial respiration and ATP levels [19, 20]. While the mechanisms underlying these phenomena have just begun to be studied, Bcl-2 has been proposed to bind complex-IV and enhance its activity [20]. In this study, we showed that Bcl-w also elevates ΔΨ_m_ and ATP levels; however, in contrast to Bcl-2, Bcl-w activated complex-I, but not complexes II-IV. While the reason for the difference between Bcl-2 and Bcl-w is not yet clear, the function of Bcl-w is consistent with the fact that complex-I is a major source of ROS in mitochondrial respiration [26]. However, in contrast to the effect of Bcl-w on complex-I activity, we found no evidence for effective binding of Bcl-w to complex I-IV, suggesting that Bcl-w activates complex-I indirectly. We indeed found that Bax directly binds to complex-I, inhibiting its activity, and that this function is prevented by Bcl-w. Considering that Bax and complex-I reside in the mitochondrial outer and inner membranes, respectively [27, 31], their interaction might be mediated by their sub-structures or subunits that protrude into or are exposed to the intermembrane space. This hypothesis is supported by the finding that Bax binds via its C-terminal tail to ND5 (Figure 9B). Considering our finding that Bak also binds to ND5 via its C-terminal tail, we propose that multidomain pro-apoptotic members are direct regulators of complex-I.

Interestingly, previous studies have shown that complex-I is frequently mutated in lung cancer patients: Nearly 40% of patients had mutations in the complex-I gene, and more than 80% of the complex-I mutations occurred in the ND5 gene [33]. This suggests mutations in complex-I, especially ND5, are critical to tumorigenesis or tumor progression, which was indeed supported by our finding that one such natural mutation in ND5 (ND5^G13289A^) promotes ROS production and cell invasion by preventing Bax/ND5 interactions. There results support the clinical relevance of Bax/ND5 interactions in cancer.

In summary, we have demonstrated the sequential interactions of cytoplasmic p53, Bcl-w, Bax and complex-I, which form a novel pathway that regulates ROS levels and invasiveness. These findings provide new insights into the regulation of cancer cell metastasis and metabolism.

## MATERIALS AND METHODS

### Antibodies and inhibitors

The following antibodies were used in this study: anti-p53 (Dako); anti-Bcl-w, anti-Bcl-X_L_, anti-Bax, anti-Bak, and anti-phospho-Akt (Cell Signaling Technology); anti-Akt, anti-ND2, anti-NDUFS1, and anti-NDUFV2 (Santa Cruz Biotechnology); anti-complex І, anti-complex II, anti-complex III, and anti-complex IV (Abcam); anti-MMP-2 (Calbiochem); anti-PI3K (Upstate Biotechnology); anti-β-actin (Sigma); anti-ND-1, and anti-ND5 (Novus Biologicals). Inhibitors of the mitochondrial respiratory chain were obtained from Sigma.

### siRNAs

All siRNAs were purchased from Ambion. When two sets of siRNAs were used, they were numbered siRNA-1 and -2. Their catalogue numbers are as follows: si-p53-1 (S605) and si-p53-2 (S606); si-Bax-1 (S1888) and si-Bax-2 (S1889); and si-Bak-1 (S1880) and si-Bak-2 (S1881). Where necessary, only the number 1 siRNAs were used and indicated without numbering, such as si-p53, si-Bax, and si-Bak. The catalogue number of si-Bcl-w was S1924.

### Cell culture and transfection

All cells were purchased from American Type Culture Collection, and were maintained in RPMI-1640 medium supplemented with 10% FBS. Expression constructs and siRNAs were introduced with Lipofectamine 2000 (Invitrogen). After 24 h recovery, the transfectants were used for experiments, as indicated. In the case of pTRE-Tight vectors, the recovered transfectants were treated with tetracycline (1 μg/mL) for an additional 16 h to induce gene expression, and then used for the specified experiments. Where indicated, transfectants were selected by treatment with G418 sulfate (0.5 mg/mL).

### Western blot analysis

Cell lysates and conditioned media were prepared using previously described methods [10]. Proteins in samples were separated by SDS-PAGE, electrotransferred to Immobilon membranes (Millipore, Bedford, MA), and analyzed using the specified antibodies and an ECL detection system (Amersham).

### Invasion assay

Cells were seeded onto the upper surfaces of Matrigel-coated polycarbonate filters (BD Biosciences) in a modified Boyden chamber (Corning), and analyzed for their invasiveness as described previously [8].

### Analysis of mitochondrial complex activities

Complex I, II, and IV activities were analyzed using the respective Microplate Assay kit (MitoSciences). Complex III activity was analyzed by incubating mitochondria (0.5 mg protein per ml), prepared using a previously described method [10] in a potassium phosphate buffer (25 mM, pH 7.2) containing 2 mM KCN and 40 μM reduced decylubiquinone. The reaction was initiated by adding 50 μM cytochrome c; reduction was measured by spectrophotometry at 550 nm.

### Measurement of cellular ATP levels

This assay was conducted by analyzing cell lysates using the ATP Bioluminescent Somatic Cell Assay Kit (Sigma-Aldrich) according to the manufacturer's instruction.

### Analysis of ROS levels

Cells were exposed to either 10 μM 2’7’-dichlorodihydrofluorescein diacetate (DCF-DA; Molecular Probes) or 10 μM MitoSOX Red (Invitrogen) for 30 min. Cell-associated levels of fluorescence were analyzed by flow cytometry.

### ΔΨm analysis

Cells were incubated with 10 μg/ml JC-1 (Sigma-Aldrich) for 30 min, and subsequently analyzed by flow cytometry. An increase in ΔΨ_m_ was monitored as a decrease in JC-1 monomers (FL-1 channel) [42].

### Co-immunoprecipitation assay

Cells were lysed in buffer containing 25 mM Tris-HCl (pH 7.4), 120 mM NaCl, 0.5% NP-40, 4 mM NaF, 100 μM Na_3_VO_4_. Equal amounts of proteins (300 μg) were incubated overnight with the indicated antibodies at 4^o^ C, followed by the addition of protein G-Sepharose beads (Amersham), and an additional 3 h incubation. The precipitates were washed three times with lysis buffer*,* and the proteins were analyzed by Western blotting.

### *In vitro* binding assay

Genes were cloned into pcDNA vectors and translated *in vitro* using TNT Quick Coupled Transcription/Translation Systems (Promega) according to manufacturer’ protocols. Protein binding was analyzed by co-immunoprecipitation. Where indicated, the expressed proteins were incubated with GST or GST-coupled proteins (Abnova), followed by precipitation with glutathione-conjugated Sepharose (Amersham). The precipitates were analyzed by Western blotting.

### Expression constructs and mutagenesis

Expression constructs were prepared using pcDNA3, pCMV/myc/mito, pEGFP-C1, and pTRE-Tight vectors. The former two vectors were obtained from Invitrogen, while the latter was from Clontech. These vectors were used for the following purposes: pEGFP-C1, for confocal microscopy and intravasation assays; pTRE-Tight, for the expression of pro-apoptotic Bcl-2 members (Bax and Bak); pCMV/myc/mito, for the expression of ND5 and ND5^G13289A^; and pcDNA3, for all other purposes. p53^R175H^, p53^K305N^, p53^K305N/R175H^, Bcl-w^G94A^, and ND5^G13289A^ were prepared using the QuikChange Site-Directed Mutagenesis Kit (Stratagene) [43].

### Animals

Female BALB/cAnNCrj-nu/nu mice (6 wks old) were purchased from Charles River. All animal experiments were performed under approved protocols of our Institutional Animal Care and Use Committee.

### Intravasation assay

H460 cells stably transfected with pEGFP-C1 vectors encoding the indicated genes were subcutaneously injected into the hind legs of mice (10^7^/mouse) to form xenograft tumors. Tumor volumes were calculated as described [44]. After 2 weeks, mice were anesthetized, blood was obtained via cardiac puncture, and 0.1 mL of blood was mixed with 2 mL RBC-lysis buffer (Intron Biotech). Cells were collected by centrifugation (350 *g*, 5 min), resuspended in PBS, stained with DAPI, and then analyzed by confocal microscopy. Circulating tumor cells were identified as GFP- and DAPI-positive cells.

### Statistical analysis

Experiments were performed three times and data are reported as means and standard deviations. Statistical significance was defined as *P* < 0.05, which was determined by a Student's *t* test or one-way ANOVA using GraphPad software.

## SUPPLEMENTARY MATERIAL AND FIGURES


