# Supplementary material for: Nuclear and cytoplasmic p53 suppress cell invasion by inhibiting respiratory Complex-I activity via Bcl-2 family proteins
Source: Oncotarget. 2014 Aug 6;5(18):8452–65. doi: 10.18632/oncotarget.2320 (PMC4226696; doi:10.18632/oncotarget.2320)
Supplement: Supplementary file 1 [file oncotarget-05-8452-s001.pdf]

Nuclear and cytoplasmic p53 suppress cell invasion by inhibiting respiratory Complex-I activity via Bcl-2 family proteins

Supplementary Material

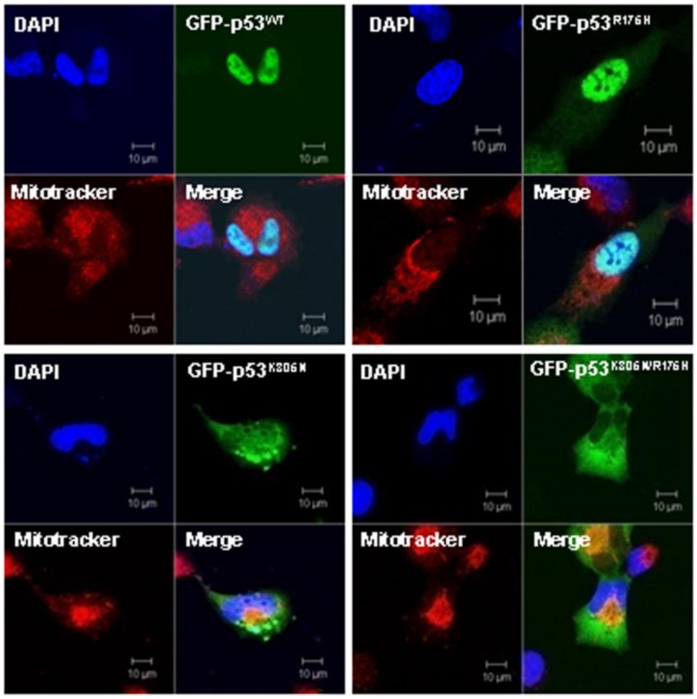

Supplementary Figure S1: Intracellular localization of wild-type and mutant p53. H1299 cells were transfected with pEGFP-C1 expression vectors for p53, p53^{R175H}, p53^{K305N}, or p53$^{K305N/R175H}$. The transfectants were stained with MitoTracker-Red and DAPI, and analyzed by confocal microscopy.

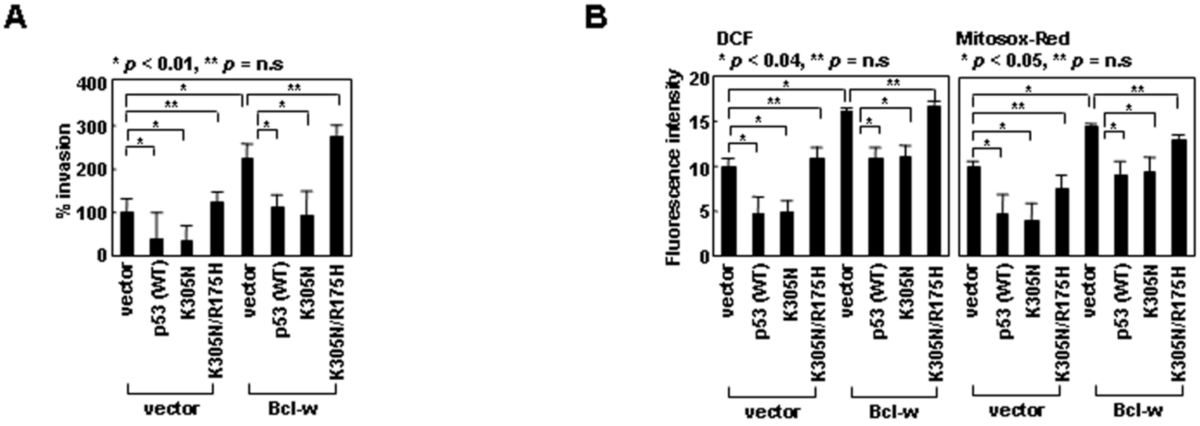

Supplementary Figure S2: Cytoplasmic p53 suppresses invasiveness and ROS production in H460 cells. (**A**) Empty pcDNA3 or vectors encoding Bcl-w, p53, $p53^{K305N}$, and $p53^{K305N/R175H}$ were introduced into H460 cells. Cellular invasiveness was assessed on Matrigel-coated filters. ns, not significant. (**B**) Transfectants were analyzed for cellular and mitochondrial ROS using DCF and MitoSOX Red probes, respectively.

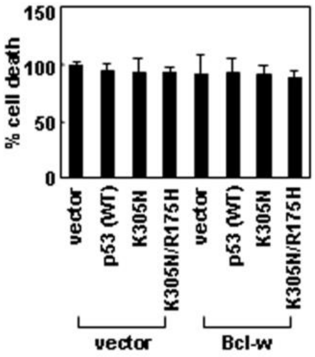

Supplementary Figure S3: The introduction of Bcl-w and/or wild-type and mutant p53 does not significantly influence the viability of H1299 cells. Empty pcDNA3 or vectors encoding Bcl-w, p53, $p53^{K305N}$, and $p53^{K305N/R175H}$ were introduced into H1299 cells in the indicated combinations. After 24 h, cells were treated with PI and viability was assessed by flow cytometry.

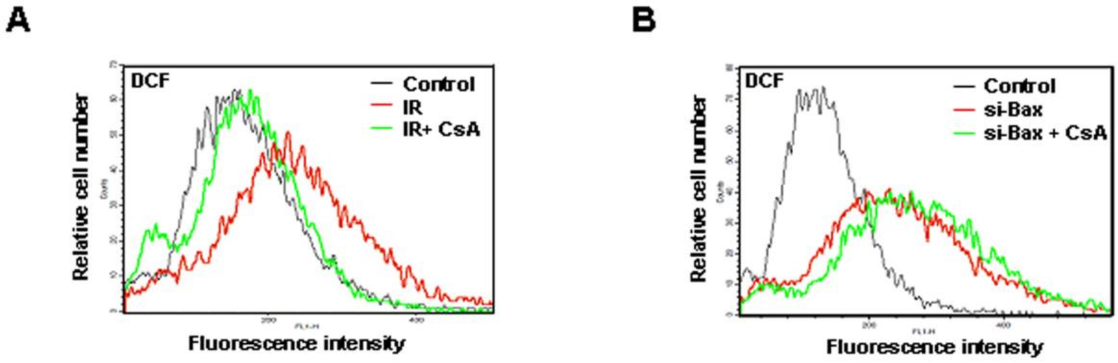

Supplementary Figure S4: Bax-knockdown promotes ROS production in an MPT-independent manner. H1299 cells were irradiated with 10 Gy γ-rays (**A**) or treated with Bax siRNA (**B**) in the presence or absence of cyclosporine A (10 μM). After 16 h, cellular ROS levels were compared by using the DCF probe.

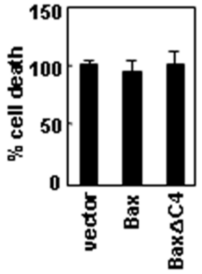

Supplementary Figure S5: Expression of Bax or BaxΔC4 does not significantly influence the viability of LoVo cells. LoVo cells were transfected with pTRE, pTRE-Bax, or pTRE-BaxΔC4 vectors. After 24 h, the cells were treated with tetracycline (1 μg/mL) for an additional 16 h to induce gene expression. Viability was assessed by flow cytometry.

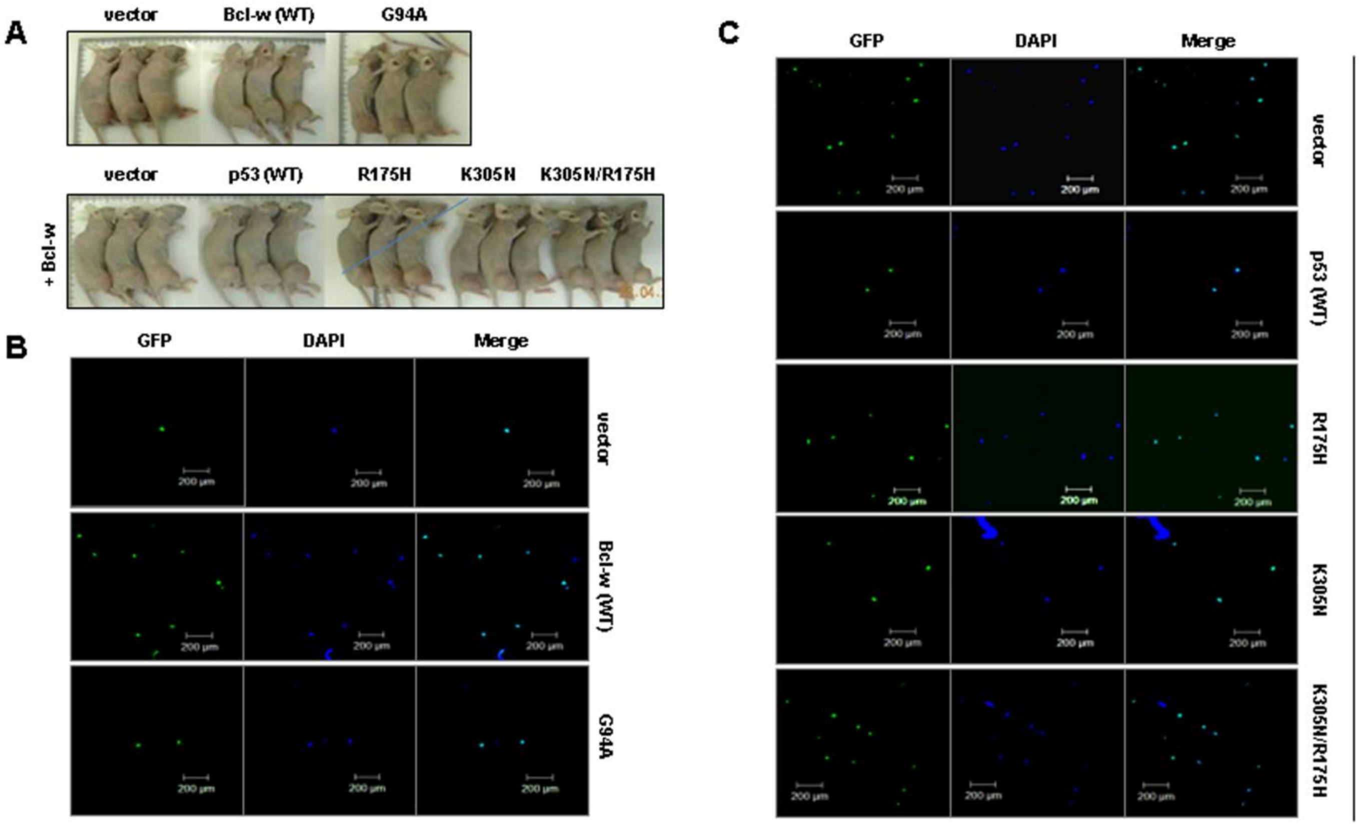

Supplementary Figure S6: Photographs and confocal images of mice and cells from the experiments described in Figure 8. (**A**) H460 cell transfectants were implanted to form xenograft tumors in mice, as described in the legend for Figure 8. Mice were photographed after 2 weeks. (**B and C**) Blood was obtained after 2 weeks. Blood cells were stained with DAPI and analyzed by confocal microscopy. Circulating tumor cells were identified as GFP- and DAPI-positive cells (azure merged images).
